# Feedback loop in miR‐449b‐3p/ADAM17/NF‐κB promotes metastasis in nasopharyngeal carcinoma

**DOI:** 10.1002/cam4.2469

**Published:** 2019-08-21

**Authors:** Qian Fei, Ming‐Yu Du, Lu‐Xi Qian, Han‐Bo Chen, Jie Chen, Hong‐Ming Zhu, Xiao‐Kang Tian, Ning Jiang, Jia‐Jia Gu, Xia He, Li Yin

**Affiliations:** ^1^ Department of Radiation Oncology The Affiliated Cancer Hospital of Nanjing Medical University & Jiangsu Cancer Hospital & Jiangsu Institute of Cancer Research Nanjing China; ^2^ Xuzhou Medical University Xuzhou China; ^3^ The Fourth Clinical School of Nanjing Medical University Nanjing China

**Keywords:** ADAM17, metastasis, miR‐449b‐3p, nasopharyngeal carcinoma, NF‐κB

## Abstract

An emerging body of evidence has promoted the understanding of the role of microRNAs (miRNAs) in tumorigenesis and progression, but the mediating function of miRNAs in nasopharyngeal carcinoma (NPC) development remains poorly elucidated. In this study, miR‐449b‐3p was downregulated in NPC specimens (*P* < .001) and cells (*P* < .05). Cytological and animal experiments provided evidence that miR‐449b‐3p inhibited NPC metastasis in vitro and in vivo. Disintegrin and metalloproteinase 17 (ADAM17) was revealed as a direct target of miR‐449b‐3p. Rescue experiments suggested that the downregulation of ADAM17 in the miR‐449b‐3p knockdown cells partially reversed the inhibition of cell invasion and migration. Luciferase reporter assay, chromatin immunoprecipitation assay, and Western blot analysis showed that ADAM17 could suppress the promoter activity and expression of miR‐449b‐3p by inducing NF‐κB transcriptional activity. In conclusion, our study provided new insights into the underlying mechanism of the invasion and metastasis of NPC. The novel miR‐449b‐3p/ADAM17/NF‐κB feedback loop could be a target for the clinical treatment of NPC.

## Graphical abstract

We demonstrated that aberrantly down‐regulated miR‐449b‐3p targeted ADAM17 to promote NPC metastasis, and ADAM17‐activated NF‐κB could transcriptionally suppress miR‐449b‐3p gene expression in turn.

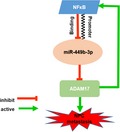

## 1 INTRODUCTION

Nasopharyngeal carcinomas (NPCs), one of the most common head and neck malignant tumors in Southeast Asia, have unique characteristics, a close association with Epstein‐Barr virus, and a high rate of metastasis.^1^ More than 70% of patients with NPC are often diagnosed at advanced stages, and this situation is associated with poor outcomes due to hidden symptoms at early stages.^2^, ^3^ Radiotherapy, in combination with chemotherapy, improves the local regional control of patients with advanced stage NPC, but distant metastasis remains the dominant cause of treatment failure.^4^, ^5^ Therefore, the rate of distant metastasis must be reduced by targeting some candidate molecules. Unfortunately, the potential molecular mechanisms of NPC metastasis are poorly understood.

MicroRNAs (miRNAs) are a class of approximately 22‐nucleotide (nt) noncoding single‐stranded RNA molecules that can negatively control gene expression in eukaryotic organisms.^6^ miRNAs are important in cancer progression, including apoptosis, proliferation, invasion, and migration.^7^ Many miRNAs, such as miR‐23a, miR‐34a, miR‐203a‐3p, and miR‐101, have been related to NPC metastasis.^8^, ^9^, ^10^, ^11^ However, searching for new molecular targets of NPC prediction and treatment remains challenging. Few studies have been conducted on the influence of the miR‐449 family on tumor metastasis. As a tumor suppressor of hepatocellular carcinoma, this family can inhibit cell migration and induce cell death,^12^ but the involvement of the miR‐449 family in NPC metastasis has yet to be studied.

Disintegrin and metalloproteinase 17 (ADAM17) is an ADAM family member that can modulate diseases and process single transmembrane proteins, such as growth factors, chemokines, cytokines, and receptors.^13^, ^14^, ^15^, ^16^ ADAM17 is overexpressed in several human tumors, such as NPC, prostate, breast, and ovarian cancers.^17^, ^18^

NF‐κB can act as a transcription factor (TF) in the progression of the cell transformation and tumorigenesis of NPC.^19^ We found via an online dataset search that two putative NF‐κB‐binding sites exist in the miR‐449b‐3p promoter. ADAM17 can activate the NF‐κB signaling pathway by promoting TNF signaling.^20^, ^21^ Thus, we hypothesized that ADAM17 might regulate the expression of miR‐449b‐3p by activating NF‐κB.

In this study, ADAM17‐activated NF‐κB negatively regulated the miR‐449b‐3p expression by binding to the miR‐449b‐3p promoter. This feedback loop in miR‐449b‐3p/ADAM17/NF‐κB might reveal a novel molecular mechanism of NPC metastasis and treatment failure.

## 2 MATERIALS AND METHODS

### 2.1 Patient samples

A total of 24 freshly frozen NPC samples (I–II stage: 6 patients; III–IV stage: 18 patients) and 4 normal nasopharyngeal epithelium samples were collected from patients who were treated in the Radiotherapy Department of Jiangsu Cancer Hospital. All tumor and normal samples were confirmed by pathologists. Before these clinical specimens were used for research purposes, the study protocol was approved by the Jiangsu Cancer Hospital's Institutional Ethical Review Board. The miR‐449b‐3p expression was explored in the data of 62 NPC samples and 6 normal nasopharyngeal epithelial samples extracted from Gene Expression Omnibus (GEO, http://www.ncbi.nlm.nih.gov/geo).

### 2.2 Cell lines

Five human NPC cell lines (6‐10B, CNE2, SUNE1, 5‐8F, and HONE1) and a human immortalized nasopharyngeal epithelial cell line (NP69) were obtained from the Jiangsu Cancer Hospital's Research Center for Clinical Oncology (Nanjing, Jiangsu, China). The culture media of human NPC cell lines were 10% calf serum (Gibco) added to RPMI‐1640 medium (Corning) at 37°C in a humidified atmosphere of 5% CO_2_. NP‐69 was propagated in a keratinocyte/serum‐free medium (Invitrogen) containing the extract of bovine pituitary (BD Biosciences) and grown at 37°C with saturated CO_2_.

### 2.3 Construction of stable cell lines overexpressing miR‐449b‐3p

The miR‐449b‐3p sequence was cloned into pGV309 vector (GeneChem). The pGV309‐449b‐3p vector or pGV309 vector (negative control; NC) was transfected into 293FT cells in accordance with the recommended protocol (GeneChem). After 48 hours of transfection, lentiviruses expressing miR‐449b‐3p (pGV309‐449b‐3p‐vector) or NC empty lenti‐vector (pGV309‐vector) were collected and used to infect NPC cells (SUNE1 and CNE2). The cells steadily overexpressing miR‐449b‐3p were selected by applying puromycin and validated through qRT‐PCR.

### 2.4 Cell transfection

SUNE1 and CNE2 cells were transfected with miR‐449b‐3p‐inhibitor/ADAM17‐siRNA in accordance with the manufacturer's protocol (RiboBio Guangzhou). Western blot analysis and qRT‐PCR (Bio‐Rad) were performed to evaluate the expression levels of miR‐449b‐3p and ADAM17 and confirm the transfection efficiency of miR‐449b‐3p‐inhibitor/ADAM17‐siRNA.

### 2.5 Cell viability and colony formation assays

Cell viability assay was conducted as previously described.^15^ A cell counting kit‐8 (Beyotime) was used to detect the growth curves of stably transfected cells. NPC cells were inoculated three times in a 96‐well plate with a density of 1.5 × 10^3^ cells per well. Absorbance was recorded at 490 nm after 24, 48, and 72 hours by using an ELX800 spectrophotometric plate reader (Bio‐Tek). For colony formation assay, the cells were plated in six‐well plates with a density of 500 cells per well and cultivated for 7‐12 days. Crystal violet staining was applied to observe the colonies. ImageJ was used to count the colonies, which were identified as the number of cells of >50.

### 2.6 Flow cytometric analysis of cell apoptosis

Stably transfected cells were seeded in six‐well plates with a density of 5‐7 × 10^5^ cells per well with three parallel holes. The cells were cultured for 48 hours, collected, and washed twice with ice‐cold phosphate buffer solution (PBS). Annexin V–FITC/PI staining was performed to detect the apoptotic cells.

### 2.7 Invasion and migration assays

Cell migration and invasion capacity were investigated by using Transwell inserts (8 µm pores; Corning) in 24‐well plates. In this procedure, 200 μL of serum‐free RPMI‐1640 medium with 1 × 10^5^ cells was added to the upper chamber with Matrigel (BD Biosciences) for invasion assay or without Matrigel for migration assay. Then, 500 μL of complete medium with 20% FBS was added to the lower chamber. The cells were cultivated for 24 or 48 hours at 37°C. Nonmigrating cells remaining at the bottom of the upper chamber were removed with cotton swabs. The cells that invaded or migrated to the bottom of the upper chamber were fixed with 4% formaldehyde and stained with crystal violet. Cells were observed and counted in five random visual fields per well under a microscope at 200× magnification. The average number of cells was calculated.

### 2.8 Wound healing assay

Wound healing assay was conducted to detect the cell migratory capacity. The NPC cells were incubated in six‐well plates for 48 hours until 90% confluency. Afterward, a 200 μL pipette tube was used to create an artificial parallel scratch, and free‐floating cells were removed with PBS. Images were captured under a light microscope at 100× magnification at 0 and 24 hours after wounding.

### 2.9 In vivo metastasis assay

A novel xenograft tumor model was constructed and spontaneous lymph node metastasis rates were detected to investigate whether or not miR‐449b‐3p inhibited NPC metastasis in vivo.^18^ In brief, 20 μL of cell suspension of 2 × 10^6^ CNE‐1 cells expressing green fluorescent protein (GFP) was injected into the footpad of athymic male mice, which were obtained from the Yangzhou University Medical Center (Yangzhou, Jiangsu, China). When the volume of the NPC xenograft tumor reached 60 mm^3^, 5 nmol miR‐449b‐3p agomir, agomir‐negative control (agomir NC), miR‐449b‐3p antagomir, or antagomir‐negative control (antagomir NC) (RiboBio) in 20 μL of saline buffer was subcutaneously injected into the plantar xenograft tumor thrice daily for 5 consecutive days. After 6 weeks, the mice were sacrificed through cervical vertebral dislocation. The mice with popliteal lymph node metastasis were counted and confirmed with GFP. Animal experimental protocols were in compliance with the Animal Committee of Nanjing Origin Biosciences, China.

### 2.10 Luciferase reporter assay

Luciferase reporter assay was applied to detect the binding between miR‐449b‐3p and ADAM17 3′‐UTR. Two other luciferase reporter plasmids (pGL3‐basic‐P1 and pGL3‐basic‐P2) were used to detect the binding of miR‐449b‐3p promoter and NF‐κB. CNE2 cells were cotransfected with 1 μg of specific plasmids by using Lipofectamine 2000 (Invitrogen). Luciferase activity after 48 hours of transfection was assessed with a dual‐luciferase reporter assay system (Promega).

### 2.11 Chromatin immunoprecipitation assay

Chromatin immunoprecipitation (ChIP) lysis buffer containing 50 mmol/L HEPES (pH 7.5), 1 mmol/L of EDTA, 1% Triton X‐100, 150 mmol/L NaCl, 0.1% sodium deoxycholate, and 0.1% SDS was added to the cells at 4°C. The DNA‐protein complexes were pulled down by using NF‐κB antibody after the 293T cells were transfected with pCMV3‐p65. The control antibody was normal rabbit IgG (CST, #2729). qPCR and DNA agarose gel electrophoresis were conducted to detect the precipitated DNA by using specific primers.

### 2.12 Western blot analysis

RIPA buffer (Beyotime) containing a protease inhibitor (phenylmethanesulfonyl fluoride) was used to lyse the treated cells and to obtain the total protein. The protein concentration was determined by using a bicinchoninicacid (BCA) protein assay kit (Beyotime), and 20 mg per protein sample was used for Western blot analysis. The following antibodies were used: monoclonal anti‐ADAM17 antibody (1:1000; Abcam, HK, ab2051), anti‐nuclear‐matrix‐p84 antibody (1:1000; Abcam, HK, ab487), anti‐E‐cadherin antibody (1:1000; CST, USA, #14472), antiNF‐κB p65 antibody (1:1000; CST, USA, #8242), anti‐vimentin antibody (1:1000; CST, USA, #5741), anti‐Snail antibody (1:2000; CST, USA,#3879), anti‐N‐cadherin antibody (1:1000; CST, USA, #13116), anti‐ZEB1 antibody (1:2000; CST, USA, #3396), anti‐phospho‐IκBα antibody (1:2000; CST, USA, #2859), anti‐β‐actin antibody (1:2000; CST, USA, #3700), and anti‐phospho‐IKKβ antibody (1:2000; CST, USA, #2697). Electrochemiluminescence detection reagent (Millipore) was applied to visualize the immunoreactive bands.

### 2.13 Immunohistochemical staining

Immunohistochemical (IHC) staining was performed to evaluate the expression of specific proteins in tissue samples from nude mice by using a manufactured kit (ZSGB‐BIO Inc). Monoclonal anti‐ADAM17 antibody was purchased from Abcam (HK, China, ab2051). Anti‐E‐cadherin antibody was obtained from CST (USA, #14472), and anti‐vimentin antibody was purchased from CST (USA, #5741).

### 2.14 NF‐κB inhibitor treatment

Caffeic acid phenethyl ester, a NF‐κB activation inhibitor, was purchased from Selleck Chemicals (S7414) and applied in accordance with the manufacturer's instruction. SUNE1 cells were incubated in six‐well plates until 90% confluency, and 10 µmol/L NF‐κB inhibitor was added to each well. After 48 hours of treatment, proteins and RNA were extracted and subjected to corresponding analyses.

### 2.15 RNA extraction and qRT‐PCR

TRIzol reagent (Invitrogen) was used to extract total RNA. Bulge‐Loop miR‐449b‐3p‐specific RT primers designed by RiboBio or ADAM17 random primers (Promega) were used for reverse transcription qRT‐PCR. An ABI7300 real‐time PCR machine (Applied Bio‐Systems) was used for qRT‐PCR. U6 or β‐actin was used as an internal control for miR‐449b‐3p or ADAM17, respectively. The primer sequences were as follows: 5′‐CTCGCTTCGGCAGCACA‐3′ and 5′‐AACGCTTCACGAATT TGCGT‐3′ for U6, 5′‐GCATTCTCAAGTCTCCACAAG‐3′ and 5′‐CCTCATTCGGGGCACATTCTG‐3′ for ADAM17, and 5′‐GGACTTCGAGCAAGAGATGG‐3′ and 5′‐AGCACTGTGTTGGCGTACAG‐3′ for β‐actin. 2^−ΔΔCt^ method was used to calculate the fold changes in ADAM17 and miR‐449b‐3p expression.

### 2.16 Statistical analysis

Statistical analyses, including ANOVA and Student's *t*‐test, were performed using GraphPad Prism 5.0 (GraphPad Software). The experiments were independently repeated thrice. Data were presented as means ± SD *P* < .05, .01, or .001 corresponded to different degrees of statistical significance.

## 3 RESULTS

### 3.1 miR‐449b‐3p is downregulated in NPC tissues and cell lines

**Figure 1:**
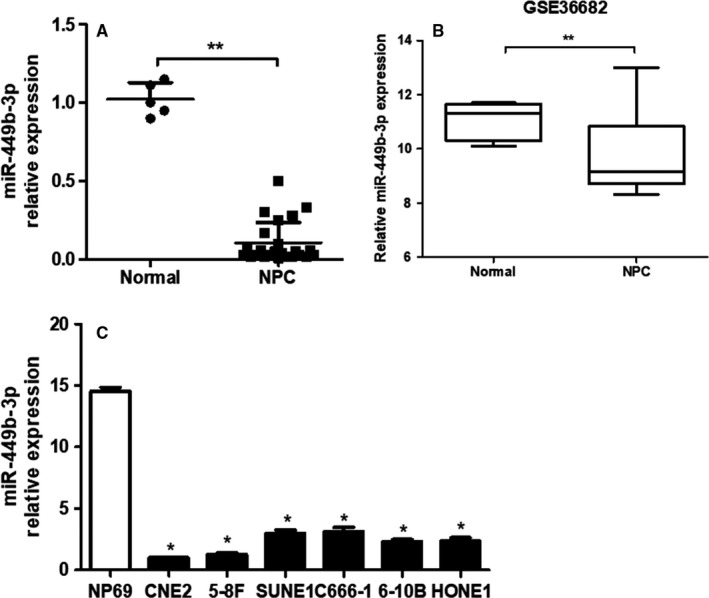
miR‐449b‐3p is downregulated in NPC tissues and cell lines. A, Relative miR‐449b‐3p expression in normal nasopharyngeal epithelial (n = 4) and NPC (n = 24) tissues. B, Data were presented as mean ± SD miR‐449b‐3p expression profile in 62 NPC samples and 6 normal nasopharyngeal epithelial samples via the GEO database. C, qRT‐PCR of the relative miR‐449b‐3p expression in NP69 and NPC cell lines. Each experiment was independently repeated at least thrice. Data were presented as mean ± SD *P* < .05 (*), .01 (**), or .001 (***) indicated different degrees of statistical significance

miR‐449b‐3p expression levels were downregulated in NPC tissues compared with those in noncancer tissues (Figure 1A, *P* < .001). This result was further confirmed with GEO database (GSE36682) (Figure 1B, *P* < .001). Similarly, NPC cell lines showed considerably lower miR‐449b‐3p expression than the immortalized nasopharyngeal epithelial cell line NP‐69 (Figure 1C). Based on the level of miR‐449b‐3p expression in six NPC cell lines, we chose CNE2 and SUNE1 cells for further study because the miR‐449b‐3p level was relative higher in SUNE1 cells and relative lower in CNE2 cells. In summary, miR‐449b‐3p was downregulated in NPC tissues and cells and may function as a tumor suppressor.

### 3.2 *miR‐449b‐3p inhibits NPC cell invasion and migration *in vitro

**Figure 2:**
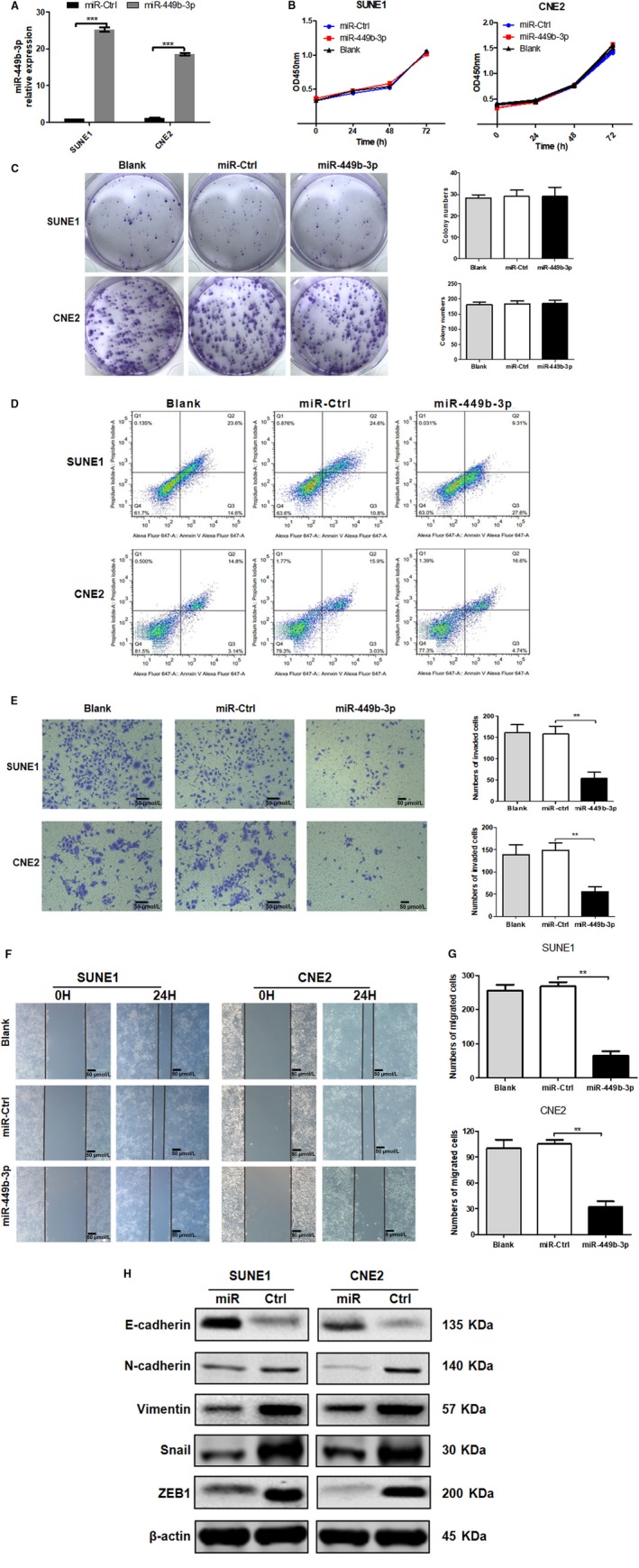
miR‐449b‐3p overexpression suppresses NPC cell invasion and migration in vitro. Stable cells overexpressing miR‐449b‐3p (miR‐449b‐3p), negative control cells (miR‐Ctrl), or the same volume of PBS (blank). A, Relative miR‐449b‐3p expression after stable cell lines were constructed. B, MTT assays were performed in stable miR‐449b‐3p‐overexpressing cells (SUNE1 and CNE2) on days 1 to 3. C, Colony formation in overexpressing miR‐449b‐3p cells (SUNE1 and CNE2) was observed through crystal violet staining. D, Flow cytometric analysis of cell apoptosis was performed in stable miR‐449b‐3p‐overexpressing cells (SUNE1 and CNE2). E, Representative images of Transwell invasion assay and cell counting results of Transwell invasion assay. F, Representative images of wound healing assay. G, Cell counting results of Transwell migration. H, Western blot analysis of EMT‐related protein expression. Each experiment was independently repeated at least thrice. Data were presented as mean ± SD *P* < .05 (*), .01 (**), or .001 (***) corresponded to different degrees of statistical significance

**Figure 3:**
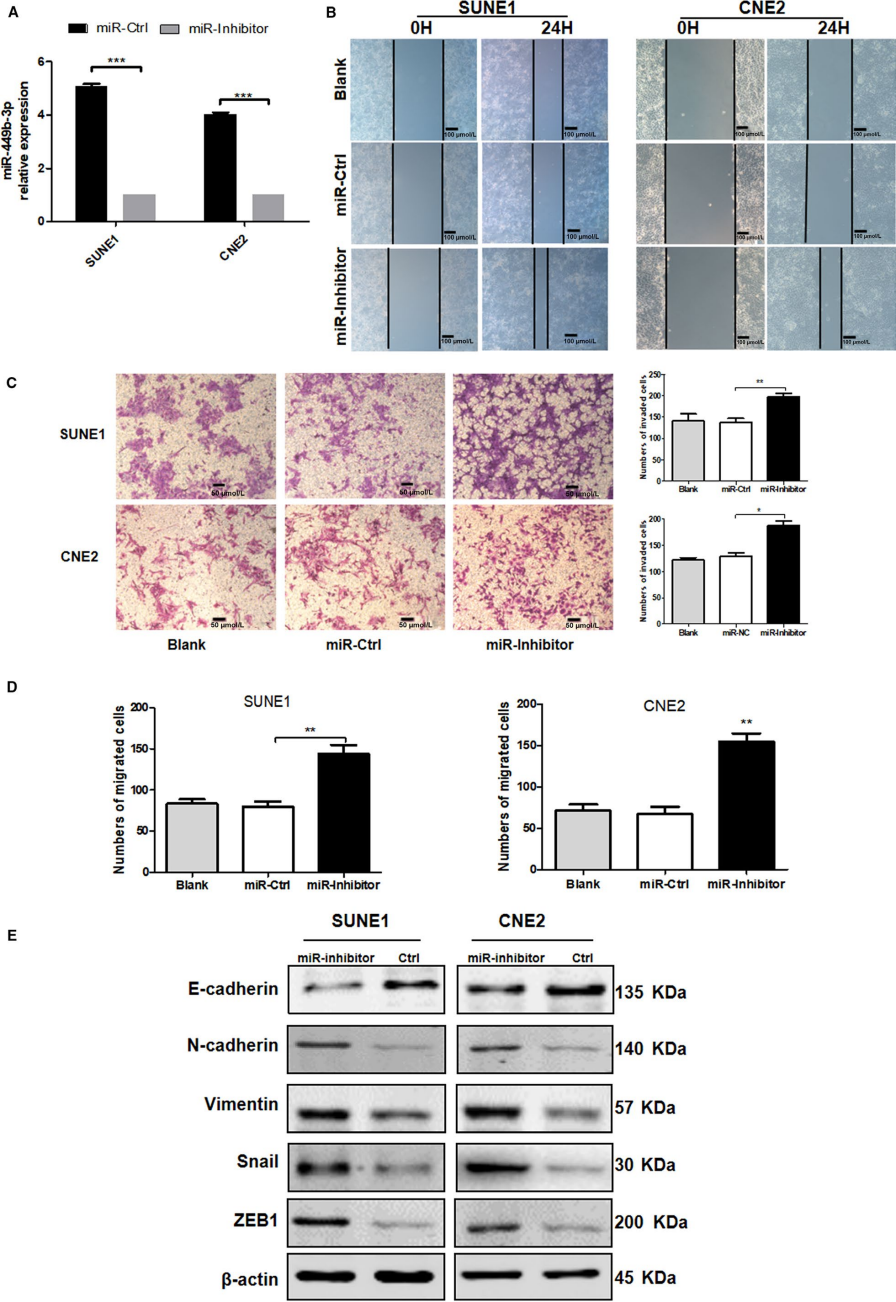
miR‐449b‐3p inhibition promotes NPC cell invasion and migration in vitro. SUNE1 and CNE2 cells were transfected with miR‐449b‐3p inhibitor (50 nmol/L), miR‐Ctrl (50 nmol/L), or the same volume of PBS (blank). A, Relative miR‐449b‐3p expression after transfection. B, Representative images of wound healing assay. C, Representative images of Transwell invasion assay, and the cell counting results of Transwell invasion assay. D, Cell counting results of Transwell migration. E, Western blot analysis of EMT‐related protein expression. Each experiment was independently repeated at least thrice. Data were presented as mean ± SD *P* < .05 (*), .01 (**), or .001 (***) corresponded to different degrees of statistical significance

CNE2 and SUNE1 cells were selected on the basis of the miR‐449b‐3p expression levels of NPC cells for further analysis. The influence of miR‐449b‐3p on cell apoptosis, proliferation, and metastasis was assessed in CNE2 and SUNE1 cells by restoring (Figure 2A) or downregulating (Figure 3A) the expression of miR‐449b‐3p in these two cell lines. The upregulated (Figure 2B‐D) or downregulated (Figure S1) miR‐449b‐3p did not significantly affect the proliferation, colony formation, and apoptosis of SUNE1 and CNE2 cells compared with those of the control groups. However, the overexpression of miR‐449b‐3p markedly inhibited NPC cell migration (Figure 2F,2) and invasion (Figure 2E). By contrast, miR‐449b‐3p inhibition caused opposite effects (Figure 3B‐D). Epithelial‐mesenchymal transition (EMT)‐related proteins were analyzed and highly associated with tumor metastasis. Western blot analysis showed that the protein expression level of E‐cadherin increased significantly in stably overexpressed miR‐449b‐3p cells, whereas Snail, vimentin, N‐cadherin, and ZEB1 levels decreased remarkably (Figure 2H). miR‐449b‐3p inhibition caused opposite effects (Figure 3E). Thus, miR‐449b‐3p could inhibit NPC cell invasion and migration.

### 3.3 miR‐449b‐3p inhibits NPC metastasis in vivo

**Figure 4:**
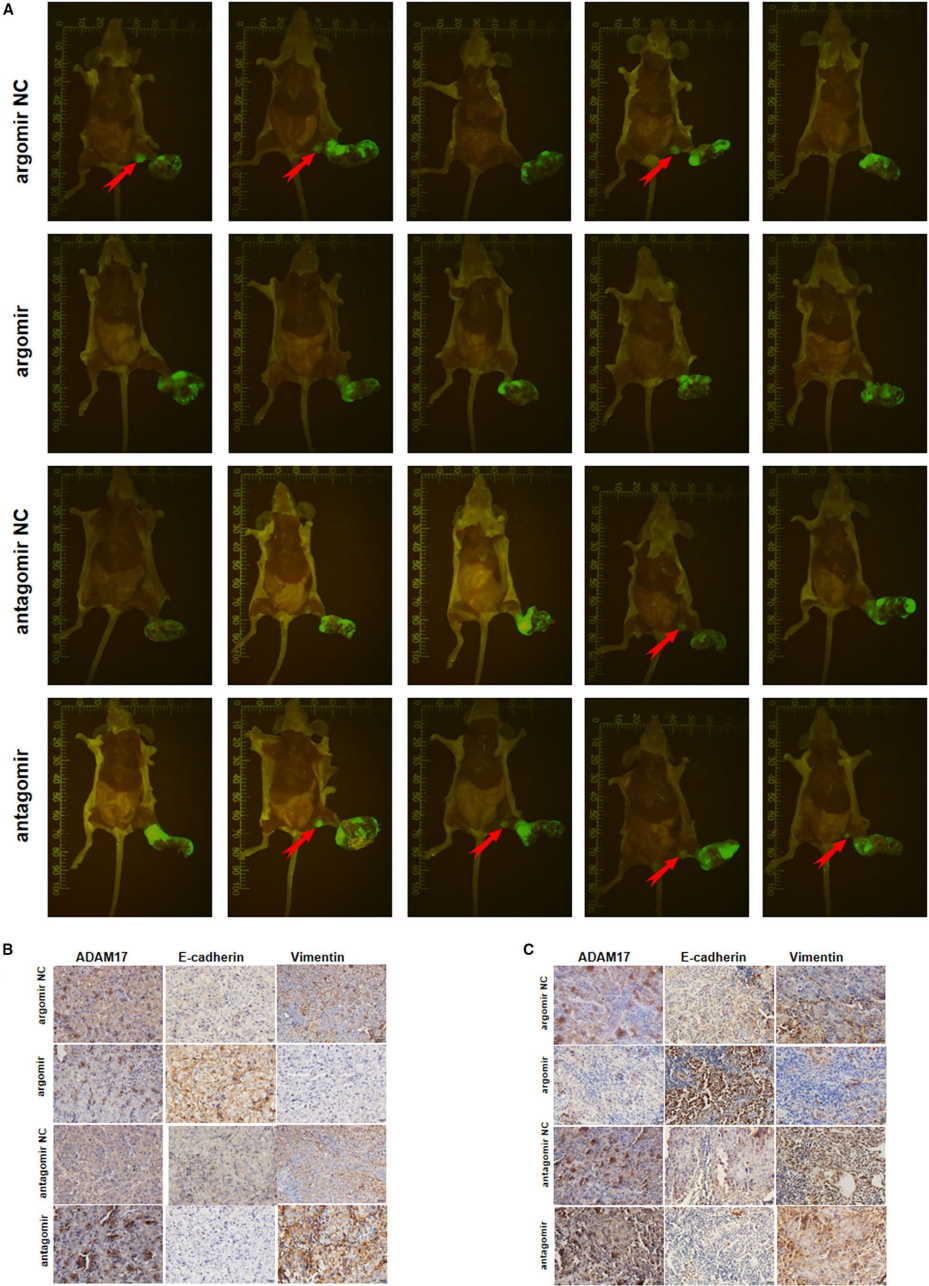
miR‐449b‐3p inhibits NPC metastasis in vivo. A, Image of xenograft tumors in miR‐449b‐3p agomir or agomir‐negative control (agomir NC) or miR‐449b‐3p antagomir or antagomir‐negative control (antagomir NC) groups of nude mice. Red arrows indicated popliteal lymph node metastasis in nude mice. B, IHC of the expression of EMT‐associated genes and ADAM17 in primary tumor. C, IHC of the expression of EMT‐associated genes and ADAM17 in metastatic tumor in lymph nodes

The influence of miR‐449b‐3p on NPC in vivo was further verified on the basis of the in vitro findings. CNE2 cells stably expressing GFP were inoculated in the feet of male nude mice to establish NPC xenograft tumor and spontaneous lymph node metastasis models (Figure 4A). miR‐449b‐3p agomir, control agomir, antagomir, or control antagomir was injected into the xenograft tumor thrice daily for 5 consecutive days until the volume of the xenograft tumor reached 60 mm^3^. Consistent with the in vitro results, the in vivo findings revealed that the number of mice with popliteal lymph node metastasis decreased in the agomir group but increased in the antagomir group compared with that in their respective controls (Figure 4A). Additionally, IHC was adopted to evaluate the expression of EMT‐associated genes, including E‐cadherin and vimentin, in primary tumor (Figure 4B) and lymph node metastatic tumor (Figure 4C). In comparison with the proteins in the respective control groups, epithelial phenotype E‐cadherin was markedly increased in the agomir group, whereas vimentin increased in the antagomir group. Thus, miR‐449b‐3p inhibited NPC metastasis in vivo.

### 3.4 ADAM17 is a direct target of miR‐449b‐3p in NPC cells

**Figure 5:**
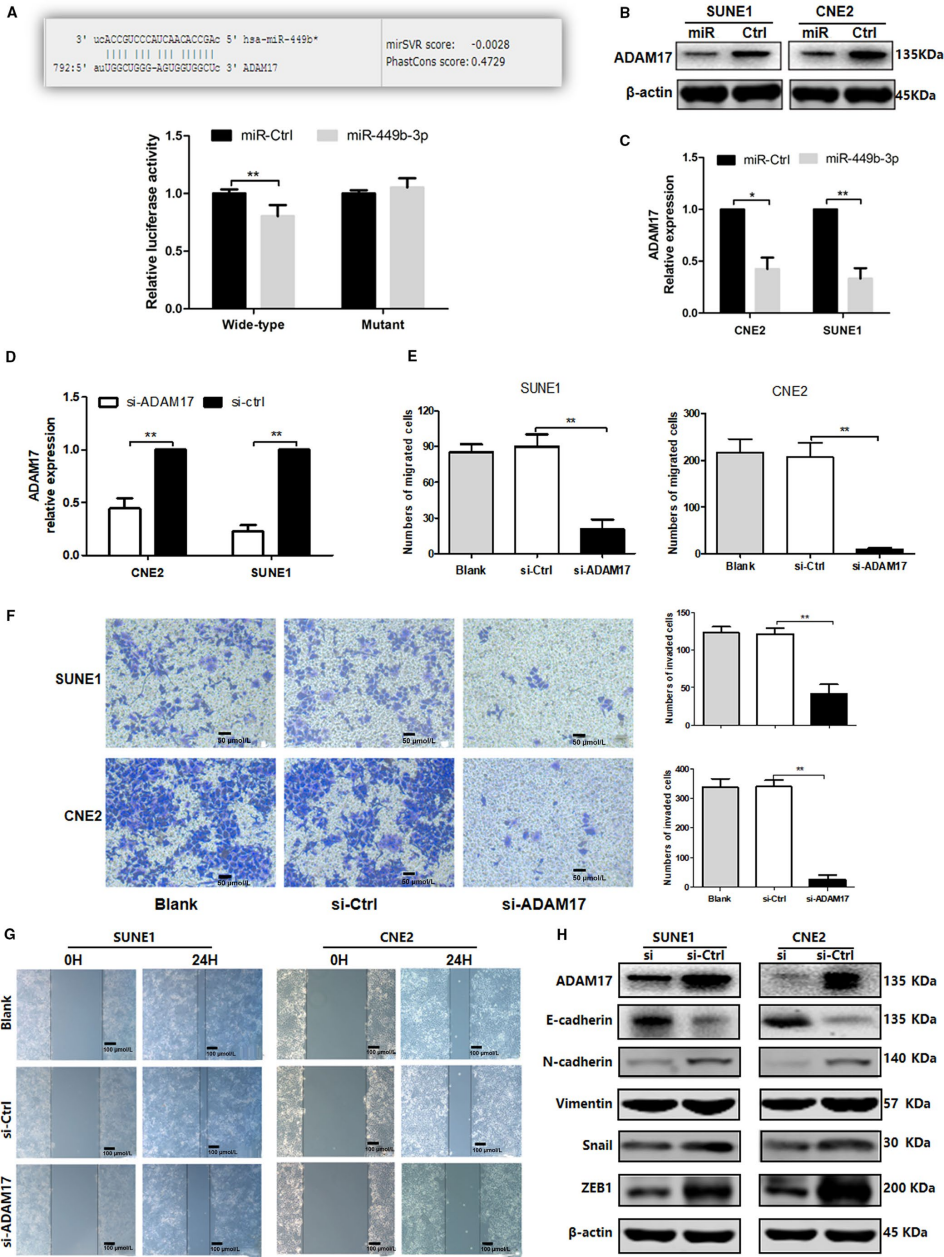
ADAM17 is a direct target of miR‐449b‐3p in NPC cells. A, ADAM17 mRNA 3′‐UTR sequences targeted by miR‐449b‐3p and luciferase reporter assay of miR‐449b‐3p and ADAM17. B, Western blot analysis of ADAM17 protein expression. C, qRT‐PCR quantification of the mRNA levels of ADAM17. D, Relative ADAM17 expression after transfectio n with ADAM17‐siRNA and negative control (si‐Ctrl). E, Cell counting results of Transwell migration. F, Representative images of Transwell invasion assay and cell counting results of Transwell invasion assay. G, Representative images of wound healing assay. H, Western blot analysis of ADAM17 and EMT‐related protein expression after transfection with ADAM17‐siRNA and negative control (si‐Ctrl). Each experiment was independently repeated at least thrice. Data were presented as mean ± SD *P* < .05 (*), .01 (**), or .001 (***) corresponded to different degrees of statistical significance

The downstream target of miR‐449b‐3p was predicted by using TargetScan6.2 to explore the mechanism underlying the inhibitory action of miR‐449b‐3p on NPC metastasis. ADAM17 was selected as the target of miR‐449b‐3p because ADAM17 was overexpressed in NPC and could promote NPC cell metastasis.^17^ Figure 5A illustrates the binding sequence between ADAM17 and miR‐449b‐3p. Luciferase reporter assay was used to validate the prediction that miR‐449b‐3p could regulate ADAM17 expression by targeting its 3′‐UTR. The luciferase activity with a normal 3′‐UTR was dramatically repressed by miR‐449b‐3p mimics, whereas the luciferase activity with a mutant 3′‐UTR was almost unaffected (Figure 5A, *P* < .01). Additionally, Western blot analysis (Figure 5B) and qRT‐PCR (Figure 5C, *P* < .05) showed that ADAM17 protein level was markedly reduced in stably miR‐449b‐3p‐overexpressing cells compared with that in miR‐Ctrl.

ADAM17‐siRNA was used to knockdown the ADAM17 expression in SUNE1 and CNE2 cells to confirm the effect of ADAM17 in NPC cell invasion and migration; qRT‐PCR and Western blot analysis were used for verification (Figure 5D and 5H). Transwell and wound‐healing assays indicated that ADAM17 silencing significantly suppressed the invasion and migration of CNE2 and SUNE1 cells (Figure 5E‐G). Western blot analysis suggested that E‐cadherin was significantly increased in the ADAM17‐siRNA group, whereas the levels of vimentin, N‐cadherin, ZEB1, and Snail were remarkably decreased (Figure 5H). In summary, these findings implied that ADAM17, as a downstream target of miR‐449b‐3p, is involved in the regulation of NPC cell metastasis.

### 3.5 ADAM17 is involved in miR‐449b‐3p‐mediated tumor metastasis

**Figure 6:**
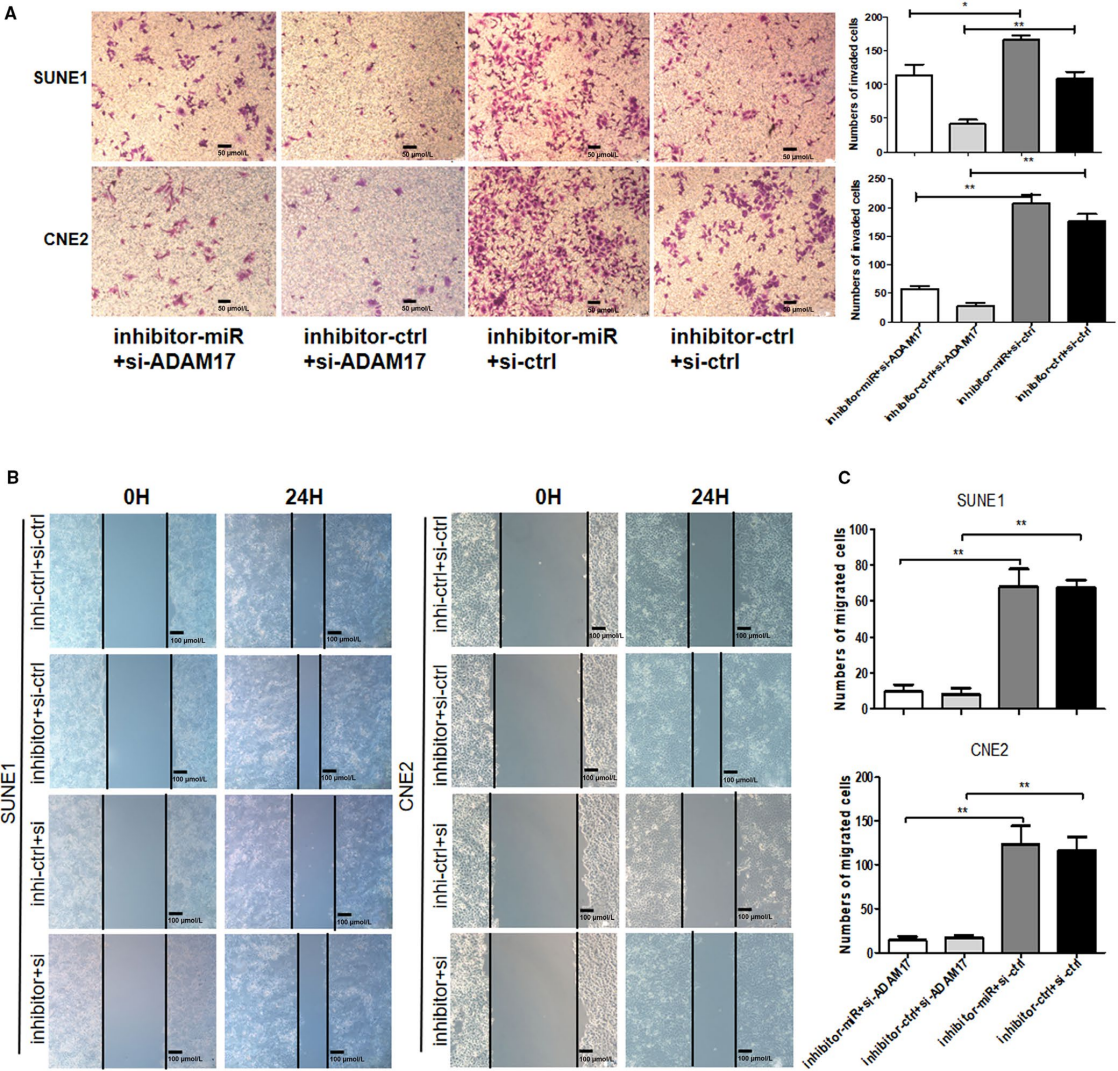
ADAM17 is involved in miR‐449b‐3p‐mediated tumor metastasis. SUNE1 and CNE2 cells were cotransfected with either miR‐449b‐3p inhibitor or negative control (inhibitor‐miR or inhibitor‐ctrl) and either ADAM17‐siRNA (si‐ADAM17) or negative control (si‐ctrl). A, Representative images and cell counting results of Transwell invasion assay. B, Representative images of wound healing assay. C, Cell counting results of Transwell migration. Each experiment was independently repeated at least thrice. Data were presented as mean ± SD *P* < .05 (*), .01 (**), or .001 (***) corresponded to different degrees of statistical significance

SUNE1 and CNE2 cells were cotransfected with either miR‐449b‐3p inhibitor or negative control and either ADAM17‐siRNA or control‐siRNA to confirm whether ADAM17 was one of the functional targets of miR‐449b‐3p. ADAM17 knockdown partially blocked the positive effects by the miR‐449b‐3p inhibitor on NPC cell invasion (Figure 6A‐C). Thus, miR‐449b‐3p mediated NPC tumor inhibition by downregulating ADAM17.

### 3.6 ADAM17‐activated NF‐κB transcriptionally inhibits miR‐449b‐3p

**Figure 7:**
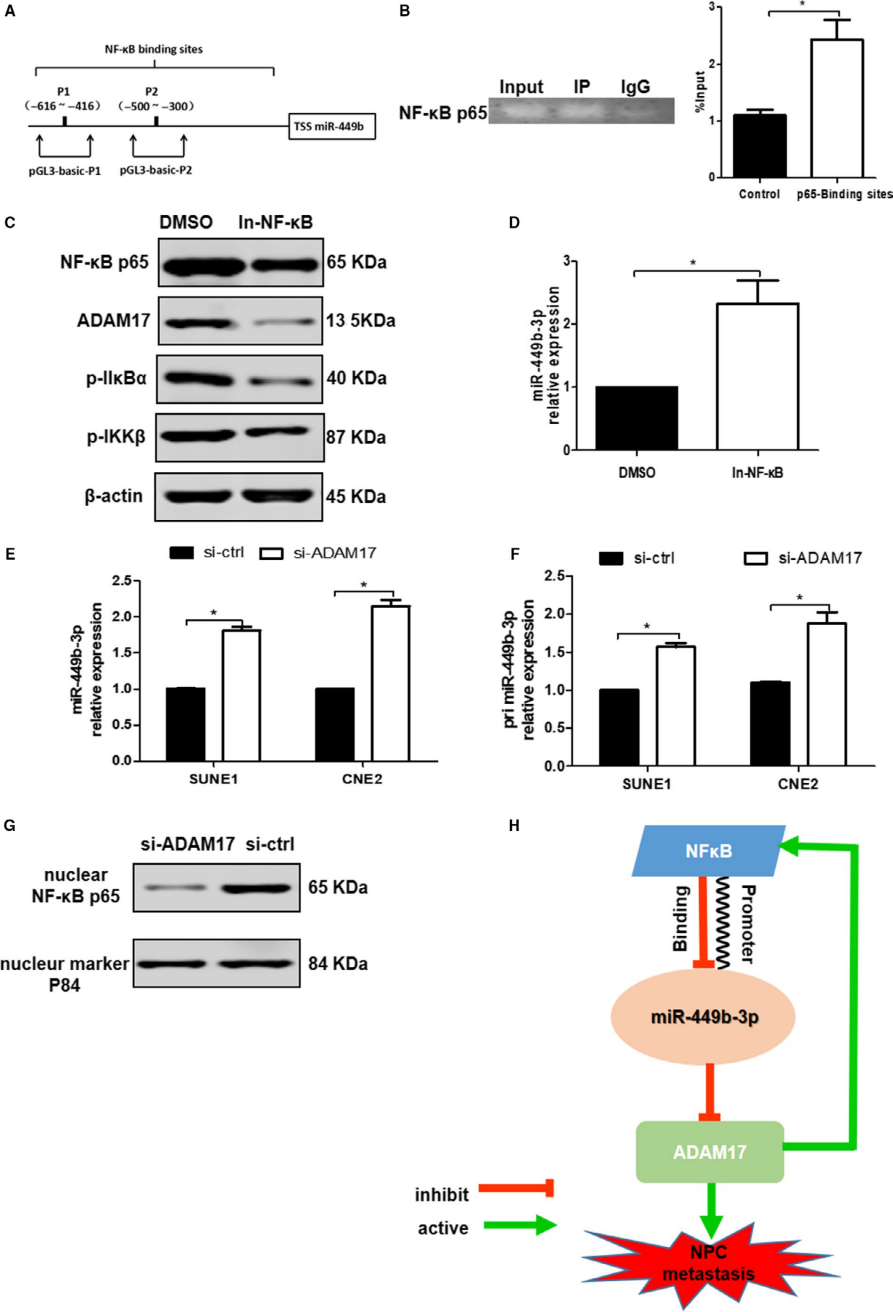
ADAM17‐activated NF‐κB transcriptionally inhibits miR‐449b‐3p. A, Two putative NF‐κB‐binding sites: −616 to −416 (P1) and −500 to −300 (P2) in the promoter region of miR‐449b‐3p. B, P1 + P2 had a binding activity with NF‐κB in the ChIP assay, which was performed by using the anti‐NF‐κB p65 antibody with IgG as a control. C, NF‐κB inhibitor (caffeic acid phenethyl ester) could inhibit NF‐κB phosphorylation, thereby blocking NF‐κB activation. D, Relative miR‐449b‐3p expression after the inhibition of NF‐κB phosphorylation. E, Relative expression of mature miR‐449b‐3p and (F) primary miR‐449b‐3p (pri miR‐449b‐3p) after ADAM17 knockdown. G, Western blot analysis of intranuclear NF‐κB p65 protein expression after ADAM17 knockdown. H, Schematic of the mechanism of ADAM17‐activated NF‐κB that negatively regulated miR‐449b‐3p expression by binding to miR‐449b‐3p promoter. Aberrantly downregulated miR‐449b‐3p‐targeted ADAM17 to promote NPC metastasis, and ADAM17‐activated NF‐κB could transcriptionally suppress miR‐449b‐3p gene expression

We investigated the upstream molecules that regulated the miR‐449b‐3p expression. Bioinformatics analysis showed that the promoter region of miR‐449b‐3p contained two putative NF‐κB‐binding sites: −616 to −416 (P1) and −500 to −300 (P2) (Figure 7A). We constructed two luciferase reporter plasmids (pGL3‐basic‐P1 and pGL3‐basic‐P2) for binding detection. Luciferase reporter assay showed that P1 and P2 were the active binding sites for NF‐κB (Figure S2). We designed P1 + P2 region for ChIP assays because P1 was close to P2. Similarly, ChIP assays showed that P1 + P2 had a binding activity with NF‐κB (Figure 7B, *P* < .05). NF‐κB inhibitor (caffeic acid phenethyl ester) can inhibit NF‐κB phosphorylation, thereby blocking NF‐κB activation.^20^, ^21^ We used the NF‐κB inhibitor to verify whether NF‐κB could influence the miR‐449b‐3p expression. Figure 7C indicated that the NF‐κB activity was successfully inhibited in NPC cells. The NF‐κB inhibitor increased the miR‐449b‐3p expression (Figure 7D, *P* < .05). ADAM17 is responsible for the activation of the NF‐κB signaling pathway,^21^, ^22^ implying that ADAM17 might regulate miR‐449b‐3p via NF‐κB transcriptional activation. Figure 7E,F show that ADAM17 silencing promoted the expression of mature miR‐449b‐3p and primary miR‐449b‐3p (pri miR‐449b‐3p) in NPC cells, thereby indicating that ADAM17 could regulate the miR‐449b‐3p expression at the transcriptional level. Additionally, the knockdown of ADAM17 reduced the intranuclear NF‐κB expression (Figure 7G). Therefore, ADAM17‐activated NF‐κB could suppress miR‐449b‐3p expression at the transcriptional level (Figure 7H).

## 4 DISCUSSION

Distant metastasis of malignant tumors, including NPC, is a challenge in oncotherapy. The metastatic feature of NPC is the main reason for its poor prognosis, and the location of metastasis involves many organs and tissues, including bone, liver, neck, lymph nodes, and intracranial region.^23^ Controlling the recurrence and distant metastasis is a key to improving the prognosis of patients with NPC. However, the molecular mechanism of NPC‐invasive metastasis remains unclear. We found a novel mechanism through which miR‐449b‐3p, ADAM17, and NF‐κB formed a feedback loop to drive NPC metastasis.

First, the microarray analysis and validation of tissue samples and cell lines revealed that miR‐449b‐3p might be involved in the carcinogenesis and progression of NPC. Bissey P A et al confirmed that the dysregulation of miR‐449b alters the TGF‐β pathway to induce cisplatin resistance by targeting TGFBI in NPC.^24^ Moreover, chemoradiotherapy‐treated patients with NPC and high miR‐449b levels showed an inferior 5‐year overall survival to those of low‐miR‐449b‐expressing patients (72.8% vs 91.8%, *P* = .017). However, a low miR‐449b‐3p expression was associated with advanced NPC stage. Patients with solid tumors are at advanced stages, considering that this phenomenon indicates that tumors have metastasized, resulting in a poor prognosis. miR‐449b‐3p may be involved in the metastasis of NPC cells. In vivo and in vitro experimental results and clinical data revealed that miR‐449b‐3p inhibited NPC metastasis. Additionally, miR‐449b‐3p did not influence proliferation, colony formation, and apoptosis of SUNE1 and CNE2 cells. The contradiction might have resulted from differences in the designs of the two studies. The sample size of the two studies is insufficient, and a study with a large sample size is needed to further verify the conclusion. miR‐449b is not synonymous to miR‐449b‐3p, and this difference may affect cell experiments.

Second, we are the first to report ADAM17 as a functional target of miR‐449b‐3p in suppressing NPC metastasis. ADAM17 is considered a molecular switch in regulating immune responses, implicated in cancer development, and overexpressed in many kinds of tumor cells.^25^, ^26^ ADAM17 can release critical protein precursor molecules, such as EGFR, growth factors, TNF‐α, and adhesion molecules, which are involved in tumor development and progression.^27^ A previous study showed that ADAM17 is upregulated in NPC cell lines compared with that in NP69 lines.^17^ Some studies have focused on targeting the regulatory relationships of miRNAs and ADAM17,^17^, ^28^, ^29^, ^30^ but few studies have examined the effect of ADAM17 on miRNAs. In the present study, ADAM17 silencing increased the expression levels of miR‐449b‐3p and primary miR‐449b‐3p. Thus, ADAM17 could modulate the expression level of miR‐449b‐3p in NPC cells at the transcriptional level.

Third, we established a feedback loop among miR‐449b‐3p, ADAM17, and NF‐κB to investigate the transcriptional regulation mechanisms of ADAM17. The NF‐κB signaling pathway can be activated by ADAM17.^21^, ^22^ The key role of NF‐κB in stimulating oncogenesis has been widely acknowledged, and carcinogenic or tumor‐promoting factors can induce the activation of NF‐κB.^31^, ^32^ Several studies have indicated that NF‐κB as a TF can regulate miRNA expression by binding to miRNA promoters in many tumor cells.^33^, ^34^, ^35^ Similarly, our study found that activated NF‐κB could inhibit the miR‐449b‐3p expression by binding to the miR‐449b‐3p promoter. Combining this result with the luciferase reporter assay of binding sites and miR‐449b‐3p upregulation caused by the inhibition of NF‐κB, we found that P1 and P2 might bind to other TFs with rejection capability. This issue should be further studied.

Several limitations existed in this study. First, this study lacked cell function tests to validate the overall feedback loop could promote NPC metastasis. Further studies should be performed to validate the function of the feedback loop. Second, the sample size of our study was insufficient. Studies with a large sample size should be carried out to further verify the conclusion. Third, this study also lacked miR‐449b‐3p‐related prognosis analysis for patients with NPC because of the short follow‐up time.

## 5 CONCLUSIONS

This study revealed that the aberrantly downregulated miR‐449b‐3p‐targeted ADAM17 promoted NPC metastasis, and ADAM17‐activated NF‐κB could transcriptionally suppress miR‐449b‐3p gene expression (Figure 7H). In theory, miR‐449b‐3p, NF‐κB inhibitor, and ADAM17 interference could be used to restrain metastasis in the clinical treatment of NPC. This study provided new insights into the mechanisms underlying the invasion and metastasis of NPC and revealed a novel regulatory loop in which miR‐449b‐3p inhibited the metastasis of NPC.

## ACKNOWLEDGMENTS

This study was supported by the National Natural Science Foundation of China (Grant number: 81672989), the National Natural Science Foundation of China (Grant number:81872192 ), the Jiangsu Provincial Commission of Health and Family Planning Young Scholars Award (Grant number: Q201501), and the Medical Young Talent Foundation of Jiangsu Provincial Health Department (Grant number: QNRC2016648).
