# Supplementary figures and images for: Feedback loop in miR‐449b‐3p/ADAM17/NF‐κB promotes metastasis in nasopharyngeal carcinoma
Source: Cancer Med. 2019 Aug 21;8(13):6049–63. doi: 10.1002/cam4.2469 (PMC6792493; doi:10.1002/cam4.2469)

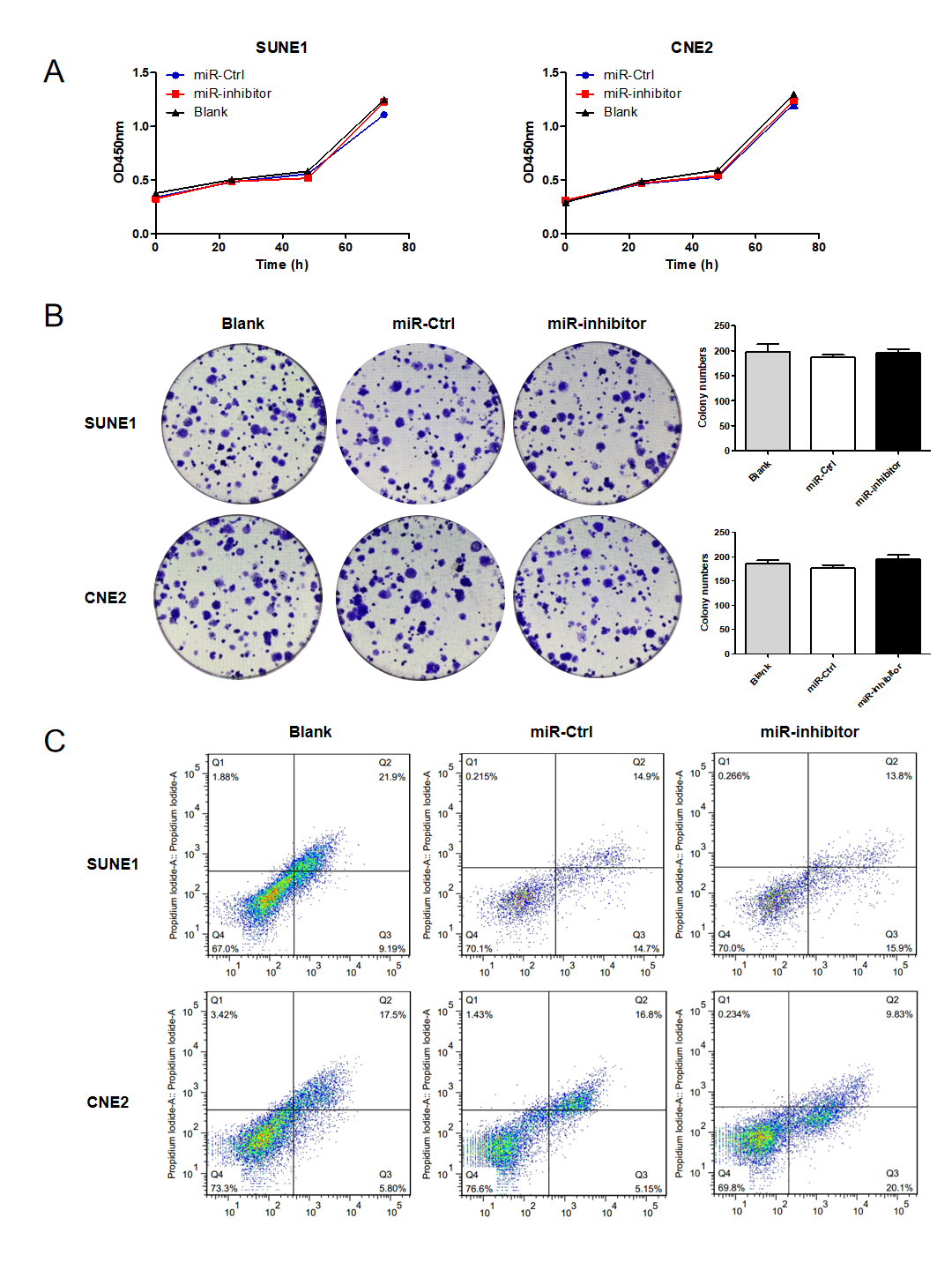

Supplement: Supplementary file 1 [file CAM4-8-6049-s001.tif]

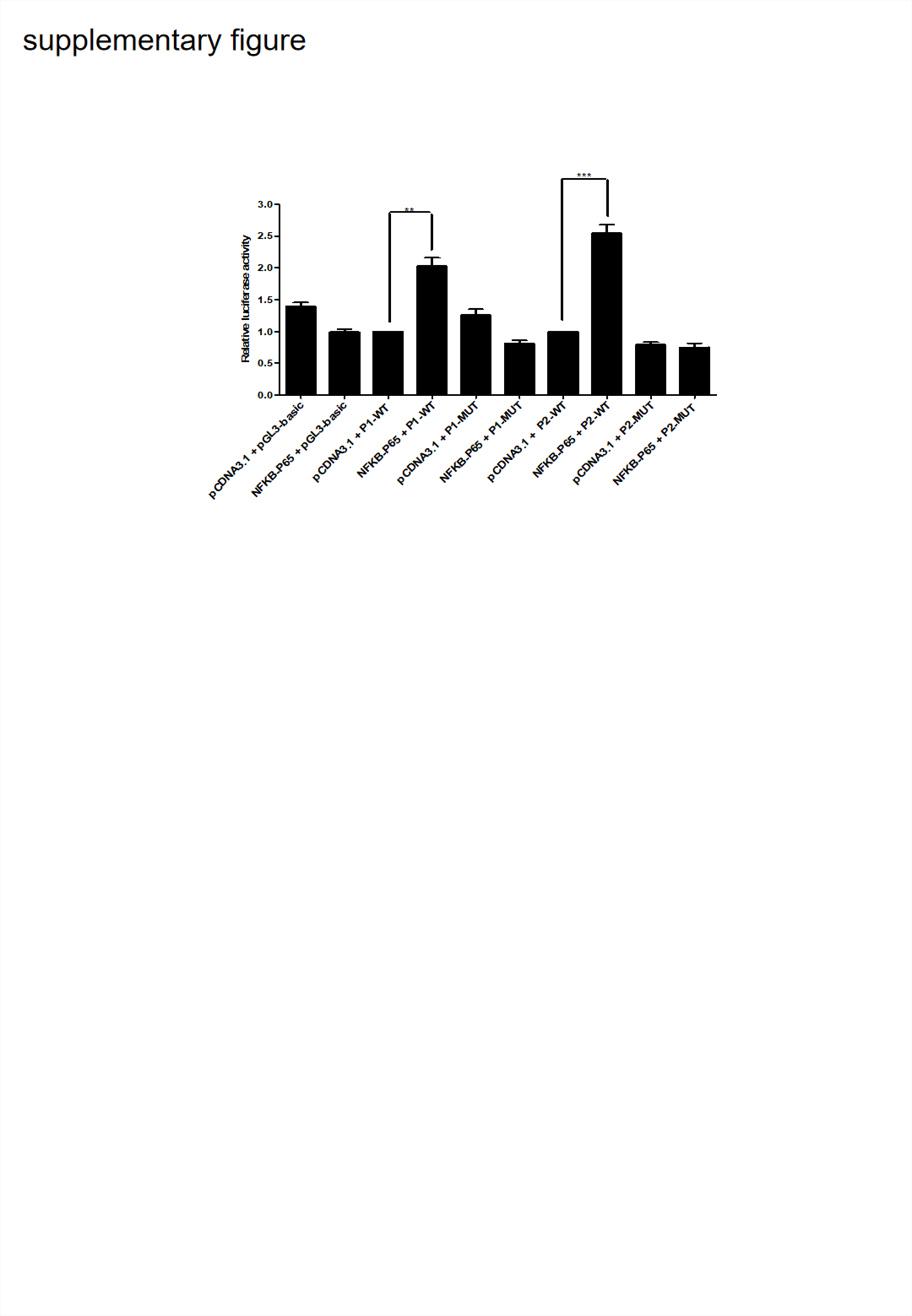

Supplement: Supplementary file 2 [file CAM4-8-6049-s002.tif]
